# Quality of life and home enteral tube feeding: a French prospective study in patients with head and neck or oesophageal cancer

**DOI:** 10.1054/bjoc.1999.0913

**Published:** 2000-01-18

**Authors:** C Roberge, M Tran, C Massoud, B Poirée, N Duval, E Damecour, D Frout, D Malvy, F Joly, P Lebailly, M Henry-Amar1

**Affiliations:** Service de Recherche Clinique, Centre François Baclesse, Route de Lion-sur-Mer, Caen cedex 5, 14076, France; Service Diététique, Centre François Baclesse, Route de Lion-sur-Mer, Caen cedex 5, 14076, France; Service de Chirurgie ORL, Centre François Baclesse, Route de Lion-sur-Mer, Caen cedex 5, 14076, France; Institut de Santé Publique, Centre René Labusquière, Bordeaux, France

**Keywords:** home enteral tube feeding, prospective study, tolerance, quality of life, cancer

## Abstract

A prospective study was conducted to evaluate the impact of home enteral tube feeding on quality of life in 39 consecutive patients treated for head and neck or oesophageal cancer at the Centre François Baclesse in Caen, France. Patients were taken as their own controls. Quality of life was evaluated using the EORTC QLQ-C30 core questionnaire, and the EORTC H&N35 and OES24 specific questionnaires. The feeding technique tolerance was evaluated using a questionnaire specifically developed for this study. Two evaluations were made, the first a week after hospital discharge (*n* = 39) and the second 3 weeks later (*n* = 30). Overall, the global health status/quality of life scale score slightly improved; among symptoms, scale scores that significantly improved (*P*< 0.05) concerned constipation, coughing, social functioning and body image/sexuality. The physical feeding technique tolerance was acceptable while the technique was psychologically less tolerated with two-thirds of the patients longing to have the tube removed. Onethird of the patients was also uncomfortable about their body image. Home enteral tube feeding was responsible for not visiting family or close relations in 15% of patients, and not going out in public in 23%. We conclude that home enteral tube feeding is a physically well accepted technique although a substantial proportion of patients may experience psychosocial distress. © 2000 Cancer Research Campaign


British Journal of Cancer (2000) **82**(2), 263–269
© 2000 Cancer Research Campaign
Article no. bjoc.1999.0913

### Quality of life and home enteral tube feeding: a French prospective study in patients with head and neck or oesophageal cancer

**C Roberge[1], M Tran[2], C Massoud[2], B Poirée[2], N Duval[2], E Damecour[2], D Frout[3], D Malvy[4], F Joly[1], P Lebailly[1] and M Henry-Amar[1]**

[1]Service de Recherche Clinique, [2]Service Diététique and [3]Service de Chirurgie ORL, Centre François Baclesse, Route de Lion-sur-Mer, 14076 Caen cedex 5, France; [4]Institut de Santé Publique, Centre René Labusquière, Bordeaux, France

**Summary** A prospective study was conducted to evaluate the impact of home enteral tube feeding on quality of life in 39 consecutive patients treated for head and neck or oesophageal cancer at the Centre François Baclesse in Caen, France. Patients were taken as their own controls. Quality of life was evaluated using the EORTC QLQ-C30 core questionnaire, and the EORTC H&N35 and OES24 specific questionnaires. The feeding technique tolerance was evaluated using a questionnaire specifically developed for this study. Two evaluations were made, the first a week after hospital discharge ($n = 39$) and the second 3 weeks later ($n = 30$). Overall, the global health status/quality of life scale score slightly improved; among symptoms, scale scores that significantly improved ($P < 0.05$) concerned constipation, coughing, social functioning and body image/sexuality. The physical feeding technique tolerance was acceptable while the technique was psychologically less tolerated with two-thirds of the patients longing to have the tube removed. One third of the patients was also uncomfortable about their body image. Home enteral tube feeding was responsible for not visiting family or close relations in 15% of patients, and not going out in public in 23%. We conclude that home enteral tube feeding is a physically well accepted technique although a substantial proportion of patients may experience psychosocial distress. © 2000 Cancer Research Campaign

**Keywords:** home enteral tube feeding; prospective study; tolerance; quality of life; cancer

The negative impact of weight loss upon morbidity and mortality of cancer patients is well known. It may decrease the response to chemotherapy as well as the tolerance to both radio and chemotherapy (Dewys et al, 1980; Vigano et al, 1994). Artificial nutrition can limit the risk of malnutrition although it is unable to restore a severely altered nutritional state (Lipman, 1991; Lopez et al, 1994; Société Française de Nutrition Entérale et Parentérale et Société Française d'Anesthésie Réanimation, 1995; Shike, 1996; Souba, 1997; Barber et al, 1998). Enteral tube feeding (ETF) is the method of choice in patients with functional digestive tract (Campos et al, 1990; Boyd and Beeken, 1994; Bozetti, 1994; Société Française de Nutrition Entérale et Parentérale et Société Française d'Anesthésie Réanimation, 1995). During short-period ETF, nasogastric tube is generally used for diet administration. Gastrostomy and jejunostomy are indicated in prolonged or permanent ETF only (Société Française de Nutrition Entérale et Parentérale et Société Française d'Anesthésie Réanimation, 1995). ETF-related complications are uncommon, the most frequent being diarrhoea (Coben et al, 1994; Société Française de Nutrition Entérale et Parentérale et Société Française d'Anesthésie Réanimation, 1995). Acute aspiration pneumonia is rare and can easily be avoided (Lopez et al, 1994).

During the last 15 years, home enteral tube feeding (HETF) has become a daily practice (Sami et al, 1990; Howard, 1993; Elia, 1995). Compared with home parenteral nutrition, HETF is a simpler and cheaper technique, with fewer related complications (Detsky et al, 1986; Elia, 1994; Howard et al, 1995). In France, HETF is not charged to the patient (i.e. it is totally reimbursed by the social security) providing that nutrition lasts at least 1 month (Ministère de la Solidarité, de la Santé et de la Protection Sociale, 1988; Ministère des Affaires Sociales, de la Santé et de la Ville, 1993).

Quality of life evaluation has become essential in all situations where the disease or its treatment are likely to induce physical, emotional, cognitive, social, family or professional impairment (Launois, 1994; Osoba, 1994; Grindel et al, 1996). Eating is not only considered a vital function but also a daily pleasure as well as a social tradition. A patient with HETF is nourished but does not eat. The meal is limited to its functional role; its social role disappears and the patient no longer gets pleasure from it. In addition, the tube can induce discomfort; it is also a reason for corporal image change. One can, therefore, speculate that these modifications interfere with patient's quality of life. In other words, it is likely that a close relationship exists between quality of life and HETF tolerance when considering that HETF intolerance can bring an end to parenteral nutrition (Société Française de Nutrition Entérale et Parentérale et Société Française d'Anesthésie Réanimation, 1995).

Quality of life and tolerance in patients with HETF have rarely been explored (Elia, 1994; Malone, 1994; Grindel et al, 1996). In studies dealing with this subject, measurements were made using non-validated instruments (Peteet et al, 1981; Rains, 1981; Nelson et al, 1986; Sami et al, 1990) or using instruments validated for

*Received 27 August 1998*
*Revised 20 July 1999*
*Accepted 2 August 1999*

*Correspondence to:* M Henry-Amar physicians (Beeken and Calman, 1994). Quality of life evaluation was restricted to physical or psychological functioning or to symptoms reported by patients (Peteet et al, 1981; Rains, 1981; Sami et al, 1990); HETF-related discomfort has been studied in hospitalized patients (Padilla et al, 1979; Bruning et al, 1988) while HETF tolerance has been mentioned only in papers dealing with personal views (Gulledge et al, 1987; Srp et al, 1989) or in reports on social and family impairments encountered by families or carers of children with HETF (Holden et al, 1991; Michaelis et al, 1992).

The aim of the present study was to evaluate the impact of HETF on quality of life in patients suffering from head and neck or oesophageal cancer, two cancers that usually necessitate enteral feeding. Our area is also the region where the incidence of these two cancers is among the highest in France (Parkin et al, 1997).

### PATIENTS AND METHODS

### Patient selection

Eligible patients fulfilled the following criteria: head and neck or oesophageal cancer; treated at the Centre François Baclesse for first line treatment or relapse; with HETF starting in the January–June 1997 period; with informed consent to participate in the study. Thirty-nine (27%) patients among the 146 patients who had enteral nutrition during the study period, were eligible for enrolment in the study. Overall, nine physicians and five dieticians participated in the study.

### Study measures

The study was conducted from January to July 1997. The French language validated self-administered questionnaire of the European Organization for Research and Treatment of Cancer (EORTC) QLQ-C30 core questionnaire (Aaronson et al, 1994) was used to appreciate generic quality of life data. The head and neck (H&N35) (Bjordal et al, 1999) and the oesophageal (OES24) (Blazeby et al, 1996) modules developed by the EORTC were added to evaluate the head and neck or oesophageal disease-targeted measures of quality of life. The QLQ-C30 core questionnaire explores six functional areas: two concern the physical aspect of functioning (physical and role functioning), three the psychosocial functioning (emotional, cognitive and social functioning), while the last one relates to quality of life in general. This questionnaire also includes a number of multi-item scales and single items assessing a range of physical symptoms (fatigue, nausea and vomiting, pain, dyspnoea, sleep disturbance, appetite loss, constipation and diarrhoea) and financial difficulties. A detailed manual for scoring procedures has been published by the EORTC (Fayers et al, 1995). For functional scales, scores computed ranged from 0 to 100, with the higher scale score representing a higher level of functioning. For item scales relative to physical symptoms and financial impact, scores computed ranged from 0 to 100, with a higher score representing a higher level of symptomatology or problems. The H&N35 and OES24 modules only include items relative to physical symptoms. They concern swallowing, pain, coughing and speech in both modules, nutritional aspects, feeling ill, social function and body image/sexuality in the H&N35 module, dysphagia, feeding difficulties, upper digestive tract disorders, emotional aspect, dry mouth and taste in the OES24 module. Two items (‘use of nutritional supplements’ and ‘use of feeding-tube’) of the H&N35 questionnaire were not analysed because all patients had a feeding-tube and nutritional supplements were never prescribed. The generic questionnaire and the specific modules addressed the patient status the week before interview. At the time of study, no questionnaire was available in French to evaluate the tolerance of HETF, as well as its family and social impact in these patients. Therefore, a second self-administered questionnaire (60 items) was specifically developed and tested prior to the study on ten patients. Most items covered in this questionnaire were objective, concerning modality of feeding technique, physical and psychological tolerance of HETF, and items relating to demographic data, family and social relationships. All items referred to the patient’s status 1 week and 4 weeks after returning home. These self-administered questionnaires (EORTC QLQ-C30 core questionnaire and specific modules, and self-developed questionnaire) were usually completed within 45 min. Clinical data were obtained from medical records. It concerned tumour location, date of diagnosis, WHO performance status, weight loss, date of start, date of end and type of initial therapy (surgery, radiation therapy, chemotherapy, combined modality), date of ETF start, clinical status at this date and previous history of HETF. Although the questionnaires were designed to be filled in by the patient, the protocol specified that a representative (CR) should always assist the patient either at home (day 7) or before or after a visit at Centre François Baclesse (day 28). However, two-thirds of patients completed the day-7 questionnaires at Centre François Baclesse because of planned follow-up visit, biologic examination or radiation therapy.

### Patient characteristics

Overall, 39 patients (38 males) were included in the study. The mean age was 58 years (range 38–74). Eighty-four per cent were married or lived as a couple. The last occupation was worker, qualified worker or employee in 77% of the patients. At the time of the study, however, 46% had retired. Patient medical characteristics are listed in Table 1. Oesophageal cancer was present in four patients only. HETF indication was first tumour care (28%) or relapse (39%) in 67% of patients; in other patients it was given because of treatment-related complication, mainly post-radiation necrosis.

### Enteral tube feeding

Nasogastric and gastrostomy tube was used in 80% and 20% of patients respectively. Nasogastric tube consisted of small bore

**Table 1** Patient medical characteristics at inclusion

| Medical characteristics (*n* = 39) | No | % |
|---|---|---|
| Tumour localization | | |
| Mouth, tongue, oropharynx | 24 | 61 |
| Hypopharynx, larynx | 11 | 29 |
| Oesophagus | 4 | 10 |
| WHO performance status | | |
| Normal or quite normal activity (code 0, 1) | 15 | 38 |
| Bedridden ≤50% of the day (code 2) | 22 | 57 |
| Bedridden > 50% of the day (code 3) | 2 | 5 |
| Weight loss | | |
| ≥10% of body weight | 23 | 60 |

*© 2000 Cancer Research Campaign*

**Table 2** EORTC QLQ-C30 and QLQ-H&N35 questionnaires for quality of life: scores at day 7 among 39 patients

| QLQ-C30 (*n* = 39) | Mean score | s.d. | H&N-35 (*n* = 35) | Mean score | s.d. |
|---|---|---|---|---|---|
| Functional scales[a] | | | | | |
| Physical functioning | 45 | 26 | | | |
| Role functioning | 55 | 38 | | | |
| Emotional functioning | 62 | 28 | | | |
| Cognitive functioning | 77 | 25 | | | |
| Social functioning | 62 | 34 | | | |
| Global health status/Qol | 45 | 19 | | | |
| Symptom scales/items[b] | | | Symptom scales/items[b] | | |
| Fatigue | 62 | 29 | Swallowing | 28 | 28 |
| Nausea and vomiting | 18 | 30 | Nutritional aspects (1) | 46 | 24 |
| Pain | 36 | 32 | Pain | 33 | 27 |
| Dyspnoea | 38 | 38 | Coughing | 38 | 35 |
| Insomnia | 38 | 38 | Feeling ill | 21 | 27 |
| Appetite loss | 37 | 45 | Social function (2) | 37 | 31 |
| Constipation | 26 | 30 | Speech | 53 | 33 |
| Diarrhoea | 26 | 28 | Body image/sexuality (3) | 40 | 32 |
| Financial difficulties | 17 | 30 | | | |

[a]Higher scores represent a higher level of functioning. [b]Higher scores represent a higher level of symptomatology or difficulty. The QLQ-H&N35 symptom scales below refer to Bjordal et al (1999). (1) This scale is composed of single items HNTE, HNOM, HNDR, HNSS + trouble eating (included in HNSO, social eating) + scale SENSES. (2) This scale is composed of SOCIAL EATING (HNSO, items 20 to 22) + scale SOCIAL CONTACT (HNSC, items 25 to 27). (3) This scale is composed of SOCIAL CONTACT (HNSC, items 18 and 28) + scale SEXUALITY (HNSX).

(4 mm) polyurethane tube. Gastrostomy tube was natural rubber latex Foley tube surgically placed. An average daily caloric intake of 2100 Kcal (Enterogil 500 Na 80®), i.e. caloric diet of 500 Kcal/500 ml) was usually delivered in intermittent nutrient intake (mean 4.4 per day, range 4–6). Additional water intake was recommended including tube rinsing after each intake (100–150 ml) and 50–100 ml if the patient felt thirsty. Seventy-seven per cent of patients had no previous experience of HETF. Education of patient and his family was given before hospital discharge by dieticians. It consisted in oral and written information during hospitalization and practical use of tube feeding the day of hospital discharge. Written information included a description of the technique, specific recommendations and advice regarding problems that can occur (i.e. thirst and hunger management, tube obstruction, diarrhoea, constipation…).

### Statistical analysis

For statistical analysis of categorical data, the Fisher exact test was used to compare independent data and the MacNemar test to compare paired data. The Kruskal–Wallis test was used to compare quantitative data. Changes between day-7 and day-28 scores were calculated as the difference between scores measured at day 28 and those measured at day 7, patients being taken as their own controls. Therefore, a positive difference of functional scores represents an improvement while a negative difference of symptom scores represents an improvement. The *STATA* and the *STATXACT* statistical software packages were used (Cytel Software Corporation, 1995; Stata Corp, 1996). Data was prospectively stored at the Clinical Research Unit of the Centre François Baclesse using a specific data management system (Wartelle et al, 1983).

### RESULTS

### Self-reported health status at day 7

Results from the EORTC QLQ-C30 core questionnaire and those from the H&N35 module are listed in Table 2. The results refer to patient status during the first week after returning home.

#### *Functional scales*

Of the six QLQ-C30 functional scales, the physical scales were scored the lowest, similar to those of global health status. None of the scales measured correlated with either tumour localization or treatment type.

#### *Symptom scales*

The nine QLQ-C30 symptom scores could be grouped into three categories. Symptoms with low impact were nausea and vomiting, constipation, diarrhoea and financial difficulties. Symptoms with intermediate impact were pain, dyspnoea, insomnia and appetite loss while fatigue was scored higher. The eight H&N35 symptom scores were in the same range as those of the QLQ-C30, with nutritional aspects and speech being associated with the higher scores. Pain score of the QLQ-C30 and that of the H&N35 questionnaires correlated ($P$ = 0.04). Social functioning score of the QLQ-C30 questionnaire and that of social function of the H&N35 questionnaire were complementary. The OES24 questionnaire was completed by the four patients with oesophageal carcinoma. Of the ten symptoms explored, five (swallowing, nutritional aspects, pain, coughing, and speech) are common to those of the H&N35 questionnaire. Scores reported by these four patients were similar to those reported by the 35 patients with head and neck cancer except for pain and coughing which were scored 0 by three patients.

© 2000 Cancer Research Campaign 

### Tolerance of HETF at day 7

The medical prescription of HETF was well followed by the patients as estimated through the number of meals (median 4) and the caloric intake (median 2100 Kcal day$^{-1}$). One-third of the patients were able to feed with a mixed diet (ETF and grinding food); these patients, however, did not display higher functional scale scores than patients who were exclusively HETF fed. Half of the patients required systematic help in setting up HETF. This help was family provided in 90% of them, and corresponded to mothering more than to a real need of physical or technical assistance.

### Physical tolerance

Digestive complaints were reported by 18–43% of patients; they were moderate and concerned nausea (18%), oesophageal reflux (33%), meteorism (33%) and wind (43%). Moderate hunger was reported by 44% of patients of whom 10% (four patients) spontaneously increased their caloric tube feeding intake. In contrast, diurnal as well as nocturnal thirst was reported by 77% of patients.

### Daily activities

HETF also induced discomfort in daily activities such as dressing (40%) or washing (54%). In addition, 25% of patients did not resume their daily activities and 20% of patients their leisure activities because of HETF.

### Psychological tolerance

Sixty-nine per cent of patients were longing to have the tube removed and 45% worried about accidental tube removal, especially during the night. One-third of patients were uncomfortable about their body image. Feeding time was felt to be too long in 51% of patients although it was similar in average to the time (45 min) they spent for lunch or dinner before the disease occurred. Sleeping disorders (in falling to sleep or accidental waking) were mentioned by 13% of patients and the same proportion reported depression since tube feeding. In these patients, the emotional functioning was significantly ($P$ = 0.022) lower scored than in patients who did not express depression.

### Changes in family and social relationships

Changes in relationships with family or close relations were reported by 13–34% of patients. They mostly concerned improvement in relationships with children (13%), spouse (28%), friends (28%) and other family members (34%). This improvement was considered as HETF-related by 7–13% of patients. On the other hand, HETF was reported as totally preventing social or family relationships in 15% of patients; it partially prevented relationships such as participating in a lunch or a dinner at children or family/friends house in 14% and 33% of patients respectively. These figures were lower when applied to lunch or dinner at patient's home (9% and 16% respectively). Finally, 8% of patients reported welcoming nobody because of HETF and 23% never went out in public.

### Changes in self-reported health status between day 7 and day 28

At day 28, 30 patients were interviewed. Of the remaining nine patients, five had died of the disease, two patients had the tube removed before day 28, one patient was rehospitalized at day 10 for a period exceeding 3 weeks, and the last patient refused the second interview. Overall results are given for these 30 patients in Table 3. Over the study period, functional scores remained unchanged or slightly improved. Similar findings were observed for symptoms (QLQ-C30) except for constipation which was significantly ($P$ = 0.02) improved. In contrast, three specific symptom scores (H&N35) significantly improved: coughing ($P$ = 0.036), social function ($P$ = 0.03) and body image/sexuality ($P$ = 0.014). No solid conclusion could be made concerning the influence of therapy on quality of life improvement since the number of patients under therapy was limited ($n$ = 5). No obvious differences existed, however, between patients with or without therapy (data not shown). The same observation applied to the following patient subgroups: patients with newly diagnosed head and neck or oesophageal cancer ($n$ = 9), patients with relapse ($n$ = 8) and tumour-free patients with tumour-related complications ($n$ = 13).

**Table 3** EORTC QLQ-C30 and H&N35 questionnaires for quality of life: variation of scores between day 7 and day 28 among 30 patients

| QLQ-C30 ($n$ = 30) | Mean difference | s.d. | H&N-35 ($n$ = 28) | Mean difference | s.d. |
|---|---|---|---|---|---|
| Functional scales[a] | | | | | |
| Physical functioning | 8 | 28 | | | |
| Role functioning | –1 | 41 | | | |
| Emotional functioning | 0 | 26 | | | |
| Cognitive functioning | –1 | 23 | | | |
| Social functioning | 5 | 26 | | | |
| Global health status/QoL | 5* | 16 | | | |
| Symptom scales/items[b] | | | Symptom scales/items[b] | | |
| Fatigue | –6 | 31 | Swallowing | 3 | 29 |
| Nausea and vomiting | 4 | 35 | Nutritional aspects | –2 | 20 |
| Pain | –4 | 28 | Pain | –5 | 18 |
| Dyspnoea | –2 | 35 | Coughing | –11** | 26 |
| Insomnia | 3 | 38 | Feeling ill | –8 | 30 |
| Appetite loss | –7 | 41 | Social function | –16** | 30 |
| Constipation | –14** | 32 | Speech | –11* | 26 |
| Diarrhoea | 5 | 39 | Body image/sexuality | –13** | 23 |
| Financial difficulties | –6 | 25 | | | |

[a]A positive difference of score = improvement of QoL. [b]A negative difference of score = improvement of QoL. *$P$ < 0.10, **$P$ < 0.05.

© 2000 Cancer Research Campaign

### Changes in tolerance of HETF between day 7 and day 28

A significant ($P = 0.016$) improvement was observed for concomitant mixed diet which was more often reported. However, it concerned liquid (five patients) and grinding solid (three patients) intake. In patients who had mixed diet at day 28, physical functioning, emotional functioning and global health quality of life scores did not significantly differ from those who had exclusive HETF. At day 28, 43% of patients reported at least one diarrhoea experience since hospital discharge. Overall, all other parameters used to estimate the physical and psychological tolerance of HETF remained unchanged over the study period.

### Concordance between the H&N35 and the HETF tolerance questionnaires

Overall, 15 items of the HETF tolerance questionnaire were a priori used to define patients who were intolerant of the technique. They concerned depression (one item), more distant family relationships (four items), HETF considered as an obstacle for going out in public (one item), for visiting close relations (one item), for welcoming home relations for a visit (one item), for receiving home or going to close relations for lunch or dinner (seven items). A patient was considered intolerant if one of the above items was mentioned at day 28. This was observed in 16 (53%) out of 30 patients. These 16 patients presented a body image/sexuality score significantly higher than that of the other patients (39.7 and 12.8 respectively; $P = 0.004$). Their social function score was also higher (28.5 and 10.4 respectively; $P = 0.055$). In contrast, no relationship was observed between intolerance of the technique and socio-demographic data, medical characteristics or feeding route (nasogastric or gastrostomy tube).

### DISCUSSION

The quality of life of patients with head and neck cancer or with oesophageal cancer is not altered as measured over a 1-month period of HETF although a substantial proportion (10–33%) of patients report that the technique represents difficulty in family and social life. Major complaints also concern diurnal and nocturnal thirst, diarrhoea, body image and the length of time before the tube is removed.

Our study is the first that prospectively assessed quality of life of cancer patients with HETF. The study was made possible because of a close participation of all individuals responsible for patient care, in particular dieticians and nurses. Three quarters of the patients completed the study including two assessments at a 3-week interval. The questionnaires used included the generic, validated quality of life core questionnaire of the EORTC (QLQ-C30) and its two specific modules on head and neck cancer (H&N35) and on oesophageal cancer (OES24) (Patrick and Deyo, 1989; Aaronson et al, 1993, 1994; Guyatt et al, 1995), and a specifically developed questionnaire aiming at evaluating the tolerance of HETF since no instrument was available. It is well established that quality of life or related measures are better assessed using self-administered questionnaires (Osoba, 1994). The HETF tolerance questionnaire, although not validated, includes items that were shown to correlate with items of the H&N35 module which might indicate that questions included in the HETF tolerance questionnaire are relevant and well understood by the patients.

The relationship between HETF and quality of life has been evaluated by interview in two studies (Nelson et al, 1986; Sami et al, 1990). Improvement or no change in quality of life as a consequence of HETF were observed in 88% (among 53 patients) and 75% (among 12 patients) of patients, including return to daily or previous professional activities in a substantial proportion of them. In these two studies, however, the proportion of patients with nocturnal HETF was not specified. In general, studies only focus on selected aspects of quality of life such as physical functioning, symptoms, or psychological impact. In 1981, Rains reported that six out of ten patients (including nine retired patients) had limited physical activities although eight maintained daily activities without the need of family help (Rains, 1981). The most frequently reported symptoms concern nose and throat soreness and dryness, and thirst (Padilla et al, 1979; Bruning et al, 1988). Our patients rarely complain of the former symptoms which can reflect a better tolerance of the material (tube) used. However, thirst remains a major symptom while digestive complaints are limited (Padilla et al, 1979). Diarrhoea is the most frequent digestive symptom but it can be avoided by increasing the time of tube feeding. Since the end of the study and to limit the proportion of patients suffering from thirst, dieticians recommend that the same amount of caloric intake be prescribed in three meals (instead of four per day) with increasing water intake between meals.

An attempt to define patients intolerant of the technique using depression or impairment in family or social relationships, ended in classifying 53% of patients as intolerant. This classification correlated well with the validated questionnaires used. Although very strict, this classification was of no help in defining an *ab initio* patient profile at risk of developing psychological intolerance to the technique.

Changes in relationships with family and friends are infrequent and when mentioned, mostly concern improvement. When an impairment is observed, it generally concerns patients who reported family problems prior to artificial feeding (Perl et al, 1980; Padilla and Grant, 1985). The psychological impact of HETF can be summarized into two aspects: the emotional response to artificial feeding and the psychological problems related to the inability to eat. The emotional response to artificial feeding depends on diagnosis and prognosis of cancer, and on personality characteristics of patients and family members involved. Peteet et al have described three emotional reactions that can be observed in patients with HETF or home parenteral nutrition: becoming more passive in demoralized patients, struggling over artificial feeding in independent patients, and an extreme preoccupation with eating and maintaining weight in very anxious patients or families who express fears about dying (Peteet et al, 1981). Most of our patients could be grouped in the first category although no information is available on their psychological characteristics. The psychological problems related to the inability to eat have been reported in patients with enteral as well as parenteral nutrition. The inability to eat is a major complaint. It is also considered by most patients as a major loss (Rains, 1981; Bruning et al, 1988). Patients report that they feel excluded from events where meals play a major part (Perl et al, 1980; Rains, 1981). They complain of their inability to taste, chew and swallow food, to drink and satisfy their appetite with certain foods (Bruning et al, 1988). It has been reported that patients do not become accustomed to having a nasogastric tube and taking food through a tube instead of through the mouth. This discomfort does not vary with

© 2000 Cancer Research Campaign time (Padilla et al, 1979). Our findings suggest, however, that only a few patients tolerate badly HETF although all have reported no change in physical discomfort over the study period. HETF resulted in depression, mentioned by the patient himself, in 27% of the patients. This proportion might be overestimated since HETF was part of the initial therapy in 67% of patients, and a consequence of complication in 33%.

All of these circumstances can induce depression per se, although depression is commonly reported (23–40%) in series of patients with head and neck cancer (Chaturvedi et al, 1996; List et al, 1997). However, depressed patients expressed an emotional functioning scale score significantly lower than that of other patients. Nasogastric tube has been reported to be far less tolerated than gastrostomy at such a level that some patients have expressed the wish to replace the former by a parenteral nutrition catheter (Srp et al, 1989). However, should the patients have the choice between the two techniques, most elderly patients would prefer HETF for two main reasons: no technical competence is required and there is less fear of technical dysfunction. While for some patients physical comfort is a priority, for others body image is essential (Srp et al, 1989). In our study, patients considered intolerant had a body image/sexuality score which altered significantly more than that of other patients, although no difference was found between patients with a nasogastric tube and those with gastrostomy in contrast to the findings of Lees (1997).

Our short-term tube feeding population study demonstrates that in patients with head and neck cancer or with oesophageal cancer, HETF is a physically well tolerated technique. Only a limited proportion of patients benefiting from this technique might need psychological support after hospital discharge.

### ACKNOWLEDGEMENTS

We express our thanks to Dr P Barrelier, A Bouvet, A Busson, J-H Jacob, Y Louis, J-M Ollivier, J-P Rame, D de Raucourt, A Rivière and A Roussel for their participation in the study, and Mrs J Macé-Lesec'h and H Costil for their technical assistance. Christophe Roberge has a Fondation pour la Recherche Médicale Research fellowship.

### REFERENCES

Aaronson NK, Ahmedzai S, Bergman B, Bullinger M, Cull A and Duez NJ (1993) The European Organization for Research and Treatment of Cancer QLQ-C30: a quality of life instrument for use in international clinical trials in oncology. *J Natl Cancer Inst* **85**: 365–375

Aaronson NK, Cull A, Kaasa S and Sprangers MA (1994) The EORTC modular approach to Quality of Life assessment in oncology. *Int J Mental Health* **23**: 75–96

Barber MD, Fearon KCH, Delmore G and Loprinzi CL (1998) Should cancer patients with incurable disease receive parenteral or enteral nutritional support? *Eur J Cancer* **34**: 279–285

Beeken L and Calman F (1994) A return to normal eating after curative treatment for oral cancer. What are the long-term prospects? *Eur J Cancer B Oral Oncol* **30B**: 367–392

Bjordal K, Hammerlid E, Ahlner-Elmqvist M, de Graeff A, Boysen M, Evensen JF, Biörklund A, de Leeuw JRJ, Fayers PM, Jannert M, Westin T and Kaasa S (1999) Quality of life in head and neck cancer patients: validation of the European Organization for Research and Treatment of Cancer quality of life questionnaire – H&N35. *J Clin Oncol* **17**: 1008–1019

Blazeby JM, Alderson D, Winstone K, Steyn R, Hammerlid E, Arraras J, Farndon JR on behalf of the EORTC Quality of Life Group (1996) Development of an EORTC questionnaire module to be used in quality of life assessment for patients with oesophageal cancer. *Eur J Cancer* **32A**: 1912–1917

Boyd KJ and Beeken L (1994) Tube feeding in palliative care: benefits and problems. *Palliat Med* **8**: 156–158

Bozetti F (1994) Is enteral nutrition a primary therapy in cancer patients? *Gut* **35** (Supp. 1): S65–S68

Bruning PF, Halling A, Hilgers FJ, Kappner G, Klein Poelhuis E, Kobashi-Schoot AM and Schonwenburg PF (1988) Postoperative nasogastric tube feeding in patients with head and neck cancer: prospective assessment of nutritional status and well-being. *Eur J Cancer Clin Oncol* **24**: 181–188

Campos AC, Butters M and Meguid MM (1990) Home enteral nutrition via gastrostomy in advanced head and neck cancer. *Head Neck* **12**: 137–142

Chaturvedi SK, Shenoy A, Prasad K, Senthilnathan S and Premlatha B (1996) Concerns, coping and quality of life in head and neck cancer patients. *Support Care Cancer* **4**: 186–190

Coben RM, Weintraub A, DiMarino AJ and Cohen S (1994) Gastroesophageal reflux during gastrostomy feeding. *Gastroenterology* **106**: 13–18

Cytel Software Corporation (1995) *StatXact3 for Windows*. Cytel Software Corporation: Cambridge, MA

Detsky AS, McLaughlin JR, Abrams HB, Whittaker JS, Whitwell J and L'abbé K (1986) A cost-utility analysis of the home parenteral nutrition program at Toronto General Hospital: 1970–1982. *J Parenter Enteral Nutr* **10**: 49–57

Dewys WD, Begg C, Lavin PT, Band PR, Bennett JM, Bertino JR, Cohen MH, Douglass HO, Engstrom PF, Ezdinli EZ, Horton J, Johnson GJ, Moertel CG, Oken MM, Perlia C, Rosembaum C, Silverstein MN, Skeel RT, Sponzo RW and Tormey DC (1980) Prognostic effects of weight loss prior to chemotherapy in cancer patients. *Am J Med* **69**: 491–497

Elia M (1994) Home enteral nutrition: general aspects and comparison between the United States and Britain. *Nutrition* **10**: 115–123

Elia M (1995) An international perspective on artificial nutritional support in the community. *Lancet* **345**: 1345–1349

Fayers P, Aaronson N, Bjordal K and Sullivan M (1995) *EORTC QLQ-C30 Scoring Manual*. EORTC Quality of Life Study Group: Brussels

Grindel GC, Whitmer K and Barsevick A (1996) Quality of life and nutritional support in patients with cancer. *Cancer Pract* **4**: 81–87

Gulledge AD, Srp F, Sharp JW, Matarese LE, O'Neill M and Steiger E (1987) Psychosocial issues of home parenteral and enteral nutrition. *Nutr Clin Pract* **2**: 183–194

Guyatt G, Feeny DH and Patrick D (1995) Measuring health-related quality of life. *Ann Intern Med* **118**: 622–629

Holden CE, Puntis JW, Charlton CP and Booth IW (1991) Nasogastric feeding at home: acceptability and safety. *Arch Dis Child* **66**: 148–151

Howard L (1993) Home parenteral and enteral nutrition in cancer patients. *Cancer* **72** (Supp. 2): 3531–3541

Howard L, Ament L, Fleming CR, Shike M and Steiger E (1995) Current use and clinical outcome of home parenteral and enteral nutrition therapies in the United States. *Gastroenterology* **109**: 355–365

Launois R (1994) La prise en compte des préférences des patients dans les choix de santé individuels et collectifs. *Rev Epidémiol Santé Publ* **42**: 246–262

Lees J (1997) Nasogastric and percutaneous endoscopic gastrostomy feeding in head and neck cancer patients receiving radiotherapy treatment at a regional oncology unit: a two year study. *Eur J Cancer Care Engl* **6**: 45–49

Lipman TO (1991) Clinical trials of nutritional support in cancer. Parenteral and enteral therapy. *Hematol Oncol Clin North Am* **5**: 91–102

List MA, Mumby P, Haraf D, Siston A, Mick R, MacCracken E and Vokes E (1997) Performance and quality of life outcome in patients completing concomitant chemoradiotherapy protocols for head and neck cancer. *Qual Life Res* **6**: 274–284

Lopez MJ, Robinson P, Madden T and Highbarger T (1994) Nutritional support and prognosis in patients with head and neck cancer. *J Surg Oncol* **55**: 33–36

Malone M (1994) Quality of life of patients receiving home parenteral or enteral nutrition support. *Pharmacoeconomics* **5**: 101–108

Michaelis CA, Warzak WJ, Stanek K and Van Riper C (1992) Parental and professional perceptions of problems associated with long-term pediatric home tube feeding. *J Am Diet Assoc* **92**: 1235–1238

Ministère de la Solidarité, de la Santé et de la Protection Sociale (1988) Circulaire n°88 – ABM 62 du 24 novembre 1988 relative aux modalités de prise en charge du matériel et des produits nécessaires à la nutrition entérale à domicile

Ministère des Affaires Sociales, de la Santé et de la Ville (1993) Circulaire DSS/AM 3 n°93 – 69 du 7 juillet 1993 relative aux modalités de prise en charge du matériel et des produits nécessaires à la nutrition entérale à domicile

Nelson JK, Palumbo PJ and O'Brien PC (1986) Home enteral nutrition: observation of a newly established program. *Nutr Clin Pract* **1**: 193–199

Osoba D (1994) Lessons learned from measuring health-related quality of life in oncology. *J Clin Oncol* **12**: 608–616

 *© 2000 Cancer Research Campaign*

Padilla GV and Grant MM (1985) Psychosocial aspects of artificial feeding. *Cancer* **55**: 301–304

Padilla GV, Grant M, Wong H, Hansen BW, Hanson RL, Bergstrom N and Kubo WR (1979) Subjective distresses of nasogastric tube feeding. *J Parenter Enteral Nutr* **3**: 53–57

Parkin DM, Whelan SL, Ferlay J, Raymond L and Young J (1997) *Cancer Incidence in Five Continents, Vol VII*. IARC Scientific Publications n°.143. IARC: Lyon, France

Patrick DL and Deyo RA (1989) Generic and disease-specific measures in assessing health status and quality of life. *Med Care* **27** (Supp. 3): S217–S232

Perl M, Hall R, Dubrick S, Englert D, Stickney S and Gardner E (1980) Psychological aspects of long-term home hyperalimentation. *J Parenter Enteral Nutr* 1980; **4**: 554–560

Peteet JR, Medeiros C and Slavin L (1981) Psychological aspects of artificial feeding in cancer patients. *J Parenter Enteral Nutr* **5**: 138–140

Rains BL (1981) The non-hospitalized tube-fed patient. *Oncol Nurs Forum* **8**: 8–13

Sami H, Saint-Aubert B, Szawlowski AW, Astre C and Joyeux H (1990) Home enteral nutrition system: one patient, one daily ration of an ‘all in one’ sterile modular formula in a single container. *J Parenter Enteral Nutr* **14**: 173–176

Shike M (1996) Nutrition therapy for the cancer patient. *Hematol Oncol Clin North Am* **10**: 221–234

Société Française de Nutrition Entérale et Parentérale et Société Française d’Anesthésie Réanimation (1995) ‘Conférence de consensus – Nutrition artificielle périopératoire en chirurgie programmée de l’adulte’. Recommandations du jury. *Ann Fr Anesth Réanim* **14** (suppl 2): 7–16

Souba WW (1997) Nutritional support. *N Engl J Med* **336**: 41–48

Srp F, Steiger E, Gulledge AD, Matarese LE, Paysinger J, Roncagli T, Stebbins J and Sullivan M (1989) Psychosocial issues of nutritional support. A multidisciplinary interpretation. *Nurs Clin North Am* **24**: 447–459

Stata Corp (1996) *Stata Statistical Software: release 5.0*. Stata Corporation: College Station, TX

Vigano A, Watanabe S and Bruera E (1994) Anorexia and cachexia in advanced cancer patients. *Cancer Surv* **21**: 99–115

Wartelle M, Kramar A, Jan P and Kruger D (1983) PIGAS: an interactive statistical database management system. In: *Proceedings of Second International Workshop on Statistical Database Management*, Hammond R and McCarthy JL (eds), pp. 124–132. National Technical Information Service, US Department of Commerce: Springfield, VA

*© 2000 Cancer Research Campaign*

## References

[PDF_00550] Aaronson N. K., Ahmedzai S., Bergman B., Bullinger M., Cull A., Duez N. J., Filiberti A., Flechtner H., Fleishman S. B., de Haes J. C. (1993). The European Organization for Research and Treatment of Cancer QLQ-C30: a quality-of-life instrument for use in international clinical trials in oncology.. J Natl Cancer Inst.

[PDF_00557] Barber M. D., Fearon K. C., Delmore G., Loprinzi C. L. (1998). Should cancer patients with incurable disease receive parenteral or enteral nutritional support?. Eur J Cancer.

[PDF_00560] Beeken L., Calman F. (1994). A return to "normal eating" after curative treatment for oral cancer. What are the long-term prospects?. Eur J Cancer B Oral Oncol.

[PDF_00563] Bjordal K., Hammerlid E., Ahlner-Elmqvist M., de Graeff A., Boysen M., Evensen J. F., Biörklund A., de Leeuw J. R., Fayers P. M., Jannert M. (1999). Quality of life in head and neck cancer patients: validation of the European Organization for Research and Treatment of Cancer Quality of Life Questionnaire-H&N35.. J Clin Oncol.

[PDF_00568] Blazeby J. M., Alderson D., Winstone K., Steyn R., Hammerlid E., Arraras J., Farndon J. R. (1996). Development of an EORTC questionnaire module to be used in quality of life assessment for patients with oesophageal cancer. The EORTC Quality of Life Study Group.. Eur J Cancer.

[PDF_00572] Boyd K. J., Beeken L. (1994). Tube feeding in palliative care: benefits and problems.. Palliat Med.

[PDF_00574] Bozzetti F. (1994). Is enteral nutrition a primary therapy in cancer patients?. Gut.

[PDF_00576] Bruning P. F., Halling A., Hilgers F. J., Kappner G., Poelhuis E. K., Kobashi-Schoot A. M., Schouwenburg P. F. (1988). Postoperative nasogastric tube feeding in patients with head and neck cancer: a prospective assessment of nutritional status and well-being.. Eur J Cancer Clin Oncol.

[PDF_00580] Campos A. C., Butters M., Meguid M. M. (1990). Home enteral nutrition via gastrostomy in advanced head and neck cancer patients.. Head Neck.

[PDF_00582] Chaturvedi S. K., Shenoy A., Prasad K. M., Senthilnathan S. M., Premlatha B. S. (1996). Concerns, coping and quality of life in head and neck cancer patients.. Support Care Cancer.

[PDF_00585] Coben R. M., Weintraub A., DiMarino A. J., Cohen S. (1994). Gastroesophageal reflux during gastrostomy feeding.. Gastroenterology.

[PDF_00589] Detsky A. S., McLaughlin J. R., Abrams H. B., Whittaker J. S., Whitwell J., L'Abbé K., Jeejeebhoy K. N. (1986). A cost-utility analysis of the home parenteral nutrition program at Toronto General Hospital: 1970-1982.. JPEN J Parenter Enteral Nutr.

[PDF_00592] Dewys W. D., Begg C., Lavin P. T., Band P. R., Bennett J. M., Bertino J. R., Cohen M. H., Douglass H. O., Engstrom P. F., Ezdinli E. Z. (1980). Prognostic effect of weight loss prior to chemotherapy in cancer patients. Eastern Cooperative Oncology Group.. Am J Med.

[PDF_00599] Elia M. (1995). An international perspective on artificial nutritional support in the community.. Lancet.

[PDF_00597] Elia M. (1994). Home enteral nutrition: general aspects and a comparison between the United States and Britain.. Nutrition.

[PDF_00603] Grindel C. G., Whitmer K., Barsevick A. (1996). Quality of life and nutritional support in patients with cancer.. Cancer Pract.

[PDF_00608] Guyatt G. H., Feeny D. H., Patrick D. L. (1993). Measuring health-related quality of life.. Ann Intern Med.

[PDF_00610] Holden C. E., Puntis J. W., Charlton C. P., Booth I. W. (1991). Nasogastric feeding at home: acceptability and safety.. Arch Dis Child.

[PDF_00614] Howard L., Ament M., Fleming C. R., Shike M., Steiger E. (1995). Current use and clinical outcome of home parenteral and enteral nutrition therapies in the United States.. Gastroenterology.

[PDF_00612] Howard L. (1993). Home parenteral and enteral nutrition in cancer patients.. Cancer.

[PDF_00617] Launois R. (1994). La prise en compte des préférences des patients dans les choix de santé individuels et collectifs.. Rev Epidemiol Sante Publique.

[PDF_00619] Lees J. (1997). Nasogastric and percutaneous endoscopic gastrostomy feeding in head and neck cancer patients receiving radiotherapy treatment at a regional oncology unit: a two year study.. Eur J Cancer Care (Engl).

[PDF_00622] Lipman T. O. (1991). Clinical trials of nutritional support in cancer. Parenteral and enteral therapy.. Hematol Oncol Clin North Am.

[PDF_00624] List M. A., Mumby P., Haraf D., Siston A., Mick R., MacCracken E., Vokes E. (1997). Performance and quality of life outcome in patients completing concomitant chemoradiotherapy protocols for head and neck cancer.. Qual Life Res.

[PDF_00628] Lopez M. J., Robinson P., Madden T., Highbarger T. (1994). Nutritional support and prognosis in patients with head and neck cancer.. J Surg Oncol.

[PDF_00630] Malone M. (1994). Quality of life of patients receiving home parenteral or enteral nutrition support.. Pharmacoeconomics.

[PDF_00632] Michaelis C. A., Warzak W. J., Stanek K., Van Riper C. (1992). Parental and professional perceptions of problems associated with long-term pediatric home tube feeding.. J Am Diet Assoc.

[PDF_00644] Osoba D. (1994). Lessons learned from measuring health-related quality of life in oncology.. J Clin Oncol.

[PDF_00648] Padilla G. V., Grant M. M. (1985). Psychosocial aspects of artificial feeding.. Cancer.

[PDF_00650] Padilla G. V., Grant M., Wong H., Hansen B. W., Hanson R. L., Bergstrom N., Kubo W. R. (1979). Subjective distresses of nasogastric tube feeding.. JPEN J Parenter Enteral Nutr.

[PDF_00655] Patrick D. L., Deyo R. A. (1989). Generic and disease-specific measures in assessing health status and quality of life.. Med Care.

[PDF_00658] Perl M., Hall R. C., Dudrick S. J., Englert D. M., Stickney S. K., Gardner E. R. (1980). Psychological aspects of long-term home hyperalimentation.. JPEN J Parenter Enteral Nutr.

[PDF_00661] Peteet J. R., Medeiros C., Slavin L., Walsh-Burke K. (1981). Psychological aspects of artificial feeding in cancer patients.. JPEN J Parenter Enteral Nutr.

[PDF_00663] Rains B. L. (1981). The non-hospitalized tube-fed patient.. Oncol Nurs Forum.

[PDF_00664] Sami H., Saint-Aubert B., Szawlowski A. W., Astre C., Joyeux H. (1990). Home enteral nutrition system: one patient, one daily ration of an "all-in-one" sterile and modular formula in a single container.. JPEN J Parenter Enteral Nutr.

[PDF_00667] Shike M. (1996). Nutrition therapy for the cancer patient.. Hematol Oncol Clin North Am.

[PDF_00673] Souba W. W. (1997). Nutritional support.. N Engl J Med.

[PDF_00674] Srp F., Steiger E., Gulledge A. D., Matarese L. E., Paysinger J., Roncagli T., Stebbins J., Sullivan M. (1989). Psychosocial issues of nutritional support. A multidisciplinary interpretation.. Nurs Clin North Am.

[PDF_00679] Vigano A., Watanabe S., Bruera E. (1994). Anorexia and cachexia in advanced cancer patients.. Cancer Surv.

